# From Protocols to Phenotypes: The Evolution of Precision Medicine in Critical Care and Its Alignment with “Choosing Wisely”

**DOI:** 10.3390/jpm16100503

**Published:** 2026-09-28

**Authors:** Raffaele Aspide, Daniele Guerino Biasucci

**Affiliations:** 1 Anesthesia and Neurointensive Care Unit, IRCCS Istituto delle Scienze Neurologiche di Bologna, 40139 Bologna, Italy; 2 Department of Clinical Science and Translational Medicine, Tor Vergata University, 00133 Rome, Italy; danielebiasucci@gmail.com

## 1. Introduction: Redefining Individualized Care

Over the last decade, the scientific literature has witnessed a conceptual shift from “personalized medicine” to “precision medicine” [1,2]. Although these terms are often used interchangeably, personalized medicine often implies a subjective tailoring of care, while precision medicine emphasizes the classification of patients into subpopulations according to biological, environmental, and lifestyle characteristics, as defined by the National Research Council [3]. In the Intensive Care Unit (ICU), this evolution is not merely semantic: it represents a move from “one-size-fits-all” bundles toward data-driven stratification [4,5]. This approach is aligned with the Choosing Wisely campaign, which promotes the avoidance of unnecessary treatments and procedures that may expose patients to harm [6].

The adoption of international guidelines has significantly improved outcomes in critical care. The Surviving Sepsis Campaign (SSC), for example, has standardized early recognition and treatment of sepsis and septic shock. However, these recommendations are derived from heterogeneous populations and provide average treatment effects that may not equally apply to every individual patient.

A useful conceptual model is to consider guidelines as the “safety floor,” ensuring minimum standards of care, while precision medicine serves as the “ceiling,” incorporating individual biological characteristics, physiological responses, comorbidities, and goals of care beyond average recommendations. The challenge in ICU practice is not to abandon guidelines, but to recognize when and how to move beyond them (Figure 1).

The increasing use of artificial intelligence (AI) in critical care represents an important clinical and ethical challenge [7]. Although AI models may achieve high predictive performance, their clinical reliability depends on appropriate development, validation, implementation, and monitoring. The ICU environment presents additional challenges because models must operate on complex, time-dependent clinical data and be integrated into time-sensitive decision-making processes. The quality of data in critical care medicine is often affected by missingness, measurement error, treatment-related confounding, and changes in clinical practice [7,8,9]. Consequently, AI systems may generate incorrect or poorly calibrated predictions when they are transferred to new settings or patient populations. Nevertheless, appropriately validated decision-support tools could assist clinicians in risk stratification and decision-making, provided that their outputs are interpreted within the clinical context [7,8,9].

In this editorial, which reflects the topics covered in the accompanying Special Issue (SI), we discuss several domains in which precision medicine may contribute to individualized critical care. These include sepsis, hemodynamic management, mechanical ventilation, neurocritical care, nutritional support, biological aging, and AI-assisted decision-making.

## 2. Sepsis: Moving Beyond the “Bundle” Paradigm

The SSC guidelines provide an essential clinical safety framework by emphasizing early recognition, timely antimicrobial therapy, source control, hemodynamic assessment, and ongoing reassessment [10]. The 2026 update also provides more detailed recommendations in several areas, including fluid management and de-escalation, underscoring the importance of adapting treatment to the patient’s evolving clinical condition [10]. At the same time, the evidence from the early goal-directed therapy era illustrates how apparently beneficial standardized strategies may change when tested across broader populations [11]. This evolution supports a more balanced and patient-centered approach rather than the rigid “one-size-fits-all” implementation of early goal-directed therapy.

Even in sepsis, we are at the dawn of a new era of clinical phenotyping. Landmark studies have identified four clinical phenotypes (α, β, γ, δ) through machine learning. These subgroups differ in severity, organ dysfunction, inflammatory profiles, and clinical outcomes [12,13]. The α-phenotype is generally associated with lower severity and better outcomes, whereas the β-phenotype is more common among older patients with comorbidities, particularly renal dysfunction. The γ-phenotype is characterized by prominent inflammation and pulmonary dysfunction, while the δ-phenotype is associated with severe shock, liver dysfunction, and the highest mortality. Importantly, these groups may exhibit different responses to interventions, including fluid strategies and vasopressor use. However, these potential differences in treatment response remain largely hypothesis-generating rather than established indications for phenotype-based treatment selection.

In this SI, Bakhshaliyeva et al. evaluated the association between oXiris^®^ therapy and hemodynamic, inflammatory, and perfusion markers in a selected population of patients with septic shock [14]. Their findings are hypothesis-generating and may indicate that some patients with severe systemic illness and renal dysfunction could derive particular benefit. However, the retrospective design, sample size, and potential for confounding require that these observations be confirmed in larger, prospective studies before the intervention can be recommended on the basis of phenotype alone.

Another important question concerns the response to steroids in sepsis. Large randomized controlled trials (RCTs), such as ADRENAL [15] and APROCCHSS [16], showed different effects of corticosteroid therapy across trials. Transcriptomic studies have suggested that biological endotypes may influence the response to corticosteroids: some molecular profiles may be associated with greater treatment benefit [17,18,19]. These observations illustrate the potential value of biological stratification, but they do not yet support routine endotype-guided corticosteroid therapy in clinical practice. Genomic tools remain impractical for routine ICU use because of time constraints and cost. Therefore, current precision approaches rely largely on surrogate markers, including clinical variables and biomarkers such as procalcitonin and lactate kinetics.

## 3. Hemodynamics and Fluid Stewardship

The old paradigm of a standard Mean Arterial Pressure (MAP) of 65 mmHg is being challenged by precision targets. The optimal blood pressure target can be influenced by chronic vascular adaptation (e.g., hypertension), microcirculatory integrity, and autoregulation thresholds. Patients with long-standing hypertension may have a right-shifted autoregulatory curve, requiring higher pressures to maintain cerebral and renal perfusion [20]. For an elderly hypertensive patient with cognitive decline, the optimal MAP target may differ substantially from 65 mmHg. A precision strategy could integrate baseline blood pressure history, cerebral perfusion monitoring (e.g., near-infrared spectroscopy), lactate clearance, urine output, and the dynamic response to vasopressors. The practical implication is not to universally increase targets, but to titrate perfusion goals based on individual tolerance and organ response.

While initial fluid resuscitation remains a cornerstone, the SSC 2026 guidelines place greater emphasis on fluid stewardship [10]. Beyond the first hours, the focus is on active de-resuscitation for patients showing signs of fluid overload. Precision medicine may facilitate this approach by identifying patients at higher risk of venous congestion, allowing clinicians to consider earlier de-resuscitation strategies when ongoing fluid administration is no longer appropriate.

Some practical examples include the comparison between macro and micro-circulation. The ANDROMEDA-SHOCK RCT demonstrated that peripheral perfusion-targeted resuscitation, assessed by capillary refill time, did not significantly reduce 28-day mortality compared with lactate-guided resuscitation, although the findings supported further investigation of peripheral perfusion as a physiologic target for resuscitation [21]. Similarly, arterial waveform analysis and machine-learning-based systems such as the Hypotension Prediction Index have been investigated as tools for anticipating hypotensive events and potentially supporting earlier intervention [22,23]. Importantly, these studies were conducted primarily in surgical or intraoperative populations, and their findings should not be directly extrapolated to septic ICU patients.

The SEPSISPAM trial showed that higher MAP targets (80–85 mmHg) did not improve overall mortality but reduced the need for renal replacement therapy in hypertensive patients [20]. However, higher targets increased arrhythmias, highlighting the trade-off between benefit and harm.

Going into more detail, in this Special Issue, Torre et al. reviewed the literature on veno-arterial extracorporeal membrane oxygenation, with a focus on monitoring systems and weaning. Despite this being a highly specialized topic, the review highlights the importance of echocardiography, arterial waveform analysis, carbon dioxide monitoring, and the usefulness of near-infrared spectroscopy for identifying physiological features that may support individualized management [24].

## 4. Acute Respiratory Distress Syndrome (ARDS) and Mechanical Ventilation

Precision medicine has transformed our understanding of ARDS, showing that it is not a single homogeneous condition. Clinical and biological studies have identified hyper-inflammatory and hypo-inflammatory sub-phenotypes with different patterns of organ dysfunction and potentially different responses to treatment [25]. The hyper-inflammatory phenotype is generally characterized by higher concentrations of inflammatory mediators, greater vasopressor requirements, more severe organ dysfunction, and worse outcomes.

The study of the pathophysiology of Ventilator-Induced Lung Injury (VILI) has taught us important lessons about prevention. The prescription of a fixed tidal volume based exclusively on predicted body weight does not adequately account for the amount of aerated lung available for tidal ventilation. In patients with a markedly reduced functional lung volume, the so-called “baby lung”, a tidal volume of 6 mL/kg of predicted body weight may still generate excessive regional stress and strain [26]. Conversely, reducing tidal volume below this value may be necessary when the available functional lung volume is particularly small, provided that gas exchange, respiratory effort, and acid–base balance remain clinically acceptable. Precision tools such as Electrical Impedance Tomography can provide bedside information about the regional distribution of ventilation and may help identify regional overdistension, derecruitment, recruitability, and the response to changes in Positive End-Expiratory Pressure (PEEP). They may therefore support a Choosing Wisely approach by discouraging excessive PEEP in patients with poorly recruitable lungs [27].

Therefore, 6 mL/kg of predicted body weight should not be regarded as a universally appropriate or intrinsically protective target. It should be considered an initial reference value within a broader physiological strategy aimed at minimizing VILI [28]. The tidal volume should subsequently be adapted according to respiratory-system mechanics, the estimated functional lung size, respiratory effort, gas exchange, and the patient’s response to changes in ventilatory settings [29].

So how can we identify phenotype-guided ventilation? Patients with recruitable lungs may benefit from higher PEEP, whereas patients with poorly recruitable lungs may develop overdistension and hemodynamic compromise. Similarly, a tidal volume that appears appropriate when normalized to predicted body weight may be excessive in relation to the patient’s functional lung size. Ventilator settings should therefore be adjusted according to integrated physiological assessment and treatment response rather than applied as fixed prescriptions. AI-driven tools can integrate ventilator waveforms and patient data to potentially support individualized ventilator management [30]. At present, however, these systems require robust external validation, transparent reporting, evaluation across different patient populations, and evidence that their use improves clinically meaningful outcomes. Rather than applying fixed settings, clinicians should aim to minimize lung stress and strain dynamically, adapting ventilation to the patient’s evolving physiology.

## 5. Decision-Making in Neurocritical Care: Timing, Indication, and Proportionality

Surgical interventions in neurocritical care—such as decompressive craniectomy, evacuation of intracranial hemorrhage, or tumor-related procedures—represent some of the most consequential decisions in the neuro ICU. While guidelines provide indications based on imaging and clinical thresholds, they often fail to fully account for patient-specific factors such as frailty, premorbid function, and long-term neurological prognosis [31].

Decompressive craniectomy for traumatic brain injury (TBI) illustrates the tension between standardized indications and individualized decision-making. Randomized trials such as RESCUEicp demonstrated a reduction in mortality but at the cost of increased rates of severe disability [32]. This highlights a central issue in precision medicine: survival benefit does not necessarily equate to meaningful recovery.

Similarly, in spontaneous intracerebral hemorrhage, the STICH II trial, which specifically enrolled patients with spontaneous supratentorial lobar intracerebral hematomas, did not demonstrate a significant overall functional benefit from early surgery compared with initial conservative treatment, although some signals of potential benefit were observed in selected patients [33]. These findings underscore the importance of phenotype-based selection, integrating radiological, clinical, and physiological variables. A precision approach to neurosurgical decision-making should include baseline functional status and frailty; radiological phenotype (e.g., lesion location, mass effect, and edema patterns); trajectory of neurological deterioration; and patient values and goals of care.

Emerging AI tools are beginning to support these decisions. Machine learning models have been developed to predict outcomes after TBI, integrating clinical and physiological data to estimate mortality and functional prognosis [34]. While not yet widely implemented, these tools may help clinicians identify patients most likely to benefit from surgery versus those at high risk of a non-beneficial intervention. Their role should currently be considered supportive rather than determinative, particularly because most available models require further external and prospective validation.

Regarding neurocritical patients, this Special Issue presents two single-center studies analyzing highly specific populations. In the study by Daian Ionel Popa et al., the authors attempt to identify the relationship between the speed of administration of a lifesaving therapy such as intravenous thrombolysis in ischemic stroke and the specific characteristics (age, body mass index, Glasgow Coma Scale, etc.) of the enrolled patient population, taking into account the specific characteristics of the center [35]. The goal, however, is to inform the implementation of current guidelines through a more individualized approach. In the study by Rustici et al., however, the authors developed the IPOGRO risk model using multivariable analysis to predict outcome in a specific population: poor-grade aneurysmal subarachnoid hemorrhage, thus potentially supporting clinical decision-making [36]. The practical implication is that neurosurgical interventions should be framed within a benefit–burden balance, where the decision to operate is not solely driven by anatomical criteria but by the likelihood of achieving outcomes aligned with patient-centered goals.

## 6. Nutritional Support: From Caloric Targets to Metabolic Precision

Nutritional therapy in critical care has traditionally been guided by standardized caloric and protein targets derived from population-based studies. Current guidelines recommend early enteral nutrition and avoidance of overfeeding, yet the optimal timing, dose, and composition remain highly variable across patients [37]. The heterogeneity of metabolic responses in critical illness—ranging from hypercatabolism to adaptive hypometabolism—limits the applicability of fixed prescriptions.

Recent evidence suggests that indirect calorimetry-guided nutrition improves the accuracy of energy delivery compared with predictive equations and may provide more physiologically appropriate nutritional targets in selected populations [38]. However, randomized data remain heterogeneous, and large trials such as EAT-ICU have not consistently demonstrated outcome benefits, highlighting that precision in measurement does not automatically translate into improved outcomes [39]. From a precision medicine perspective, nutritional strategies should incorporate dynamic assessment of energy expenditure, phase of illness (early acute versus recovery), degree of inflammation and organ dysfunction, and gastrointestinal tolerance.

Importantly, early full feeding in all patients may be harmful. Permissive underfeeding or trophic feeding during the acute phase has shown comparable outcomes with fewer complications in randomized trials [40]. Thus, a pragmatic approach is to avoid early overcorrection of caloric deficits, particularly in unstable patients.

That said, in the article by Gremese et al., the authors explore inflammatory biomarkers and the associated mass loss in critically ill patients in the ICU [41]. Their findings further illustrate the potential value of integrating inflammatory trajectories with changes in body composition when considering individualized nutritional strategies. In the not-too-distant future, it may be possible to personalize nutritional therapy by collecting relatively simple clinical, biochemical, and body-composition data during the early stages of critical illness.

AI may further refine nutritional support by integrating metabolic, biochemical, and clinical data to predict individual energy needs and tolerance. Early machine learning models have demonstrated the ability to estimate resting energy expenditure using approaches that may complement conventional predictive equations [42]. The clinical implication is that nutrition should not be viewed as a protocolized intervention but as a dynamic, titratable therapy, where the goal is not to achieve theoretical targets at all costs, but to align metabolic support with the patient’s evolving physiological state.

## 7. Age: A Potentially Valuable Parameter in Precision Medicine

Precision medicine is undergoing a profound paradigm shift: the traditional approach based on identifying exclusively genomic alterations is giving way to a more holistic view of the patient, in which biological age may provide additional information about functional reserve and systemic heterogeneity. While chronological age merely records the passage of time, biological age may capture aspects of physiological aging that are not fully reflected by chronological age.

Recent studies illustrate the potential of biological aging measures to contribute to risk stratification. A landmark study published in Nature Medicine by Tian et al. investigated the association between biological aging and generational shifts in early-onset cancer risk. In a large UK Biobank analysis, greater systemic biological aging was associated with higher risk of early-onset solid cancers, independent of genetic risk measures [43]. In parallel, research published in Nature Medicine by Ding et al. [44] demonstrated that plasma proteomic signatures may capture aspects of cellular aging and were associated with disease status, incident disease, and mortality in large population cohorts. The integration of such “biological clocks” into clinical practice therefore represents a promising research avenue for more individualized risk assessment and prevention strategies, although their clinical utility in critically ill patients remains to be established.

Our SI contains an excellent analysis of the available literature on stratification scores in emergency departments. Maccauro et al. highlighted the predictive limitations of traditional scores in patients > 65 years of age, emphasizing the need to use more up-to-date scores, which include measures related to biological age and may incorporate AI-based models [45]. Ithamar Cheyne et al. also address the topic of “Deep Biological Clocks” by analyzing the literature in a scoping review: beyond age, is it possible to identify a “phenotype” of a critically ill patient and correlate it with a specific outcome [46]? The available evidence is promising, but prospective validation is still required before biological aging measures can be incorporated into routine ICU prognostication or treatment decisions.

## 8. AI, Big Data, and the “Choosing Wisely” Philosophy

The integration of Electronic Health Records (EHRs) and AI-driven decision-support systems provides new opportunities to support more selective and individualized care in the ICU [7,8,9]. However, the current literature indicates that most ICU AI models remain at the development or validation stage, with relatively limited prospective clinical integration [9]. Several machine learning models are already in clinical or near-clinical use. Regarding antibiotic stewardship, rapid molecular diagnostics, including PCR- and MALDI-TOF-based approaches, may support earlier identification of pathogens and more targeted antimicrobial therapy. Such strategies fit within the broader principles of antibiotic stewardship by facilitating de-escalation when clinically appropriate [47]. Precision medicine approaches in sepsis similarly seek to identify patient subgroups that may respond differently to targeted therapies [48], although prospective validation remains essential before routine clinical implementation. Still in the context of sepsis, we can include sepsis prediction algorithms such as TREWS, which analyze EHR data to detect early deterioration and support earlier clinical recognition [49].

Currently, much of the effort in AI development is focused on its predictive capabilities. The AI Clinician, for example, used reinforcement learning to identify treatment strategies for sepsis that differed from observed physician practice in retrospective data [50]. Such approaches are potentially informative but should not be interpreted as evidence that AI-generated treatment recommendations outperform clinicians in prospective clinical practice. Reinforcement learning in healthcare also faces substantial methodological challenges, including confounding, selection bias, and the need for rigorous prospective evaluation [51]. AI systems face several challenges, including lack of interpretability (“black box” models), risk of bias in training data, limited external validation, and the potential for automation bias. Thus, AI should be viewed as an augmentation tool, supporting but not replacing clinical reasoning.

## 9. Conclusions

The evolution from personalized to precision medicine in critical care reflects a shift from average-based to individualized decision-making. Guidelines remain essential, providing a safety framework that ensures minimum standards of care. However, they should not be interpreted as rigid prescriptions that define the limits of treatment. The practical question for clinicians is not whether precision medicine is desirable, but how to implement it without increasing complexity or harm.

A pragmatic framework for precision critical care may include five principles: (a) start from guidelines (safety floor): ensure that every patient receives evidence-based standard care; (b) assess individual context: consider comorbidities, frailty, and baseline physiology; (c) use dynamic data: monitor response to interventions rather than relying exclusively on static thresholds; (d) incorporate decision-support tools: use AI outputs critically, integrating them with clinical judgment; and (e) reassess continuously: precision medicine is an iterative process rather than a one-time classification.

Precision medicine does not justify doing more. Instead, it should help identify patients unlikely to benefit from aggressive interventions, situations where less invasive strategies are preferable, and points at which escalation becomes non-beneficial or disproportionate. Future research should focus on prospective validation of phenotype-guided interventions, clinically usable multimodal tools, transparent and equitable AI systems, and outcomes that include functional recovery, quality of life, and patient-centered goals. Importantly, success will depend not only on technological innovation but also on clinical usability and interpretability. Precision medicine, supported by physiological insight, phenotype recognition, and AI, offers the opportunity to refine care, tailoring interventions to those most likely to benefit. The ultimate goal is not to increase the number of interventions, but to improve their appropriateness, timing, and proportionality. In this paradigm, guidelines define the floor, but clinical intelligence, augmented by data, determines how high we should go.

## Figures and Tables

**Figure 1:**
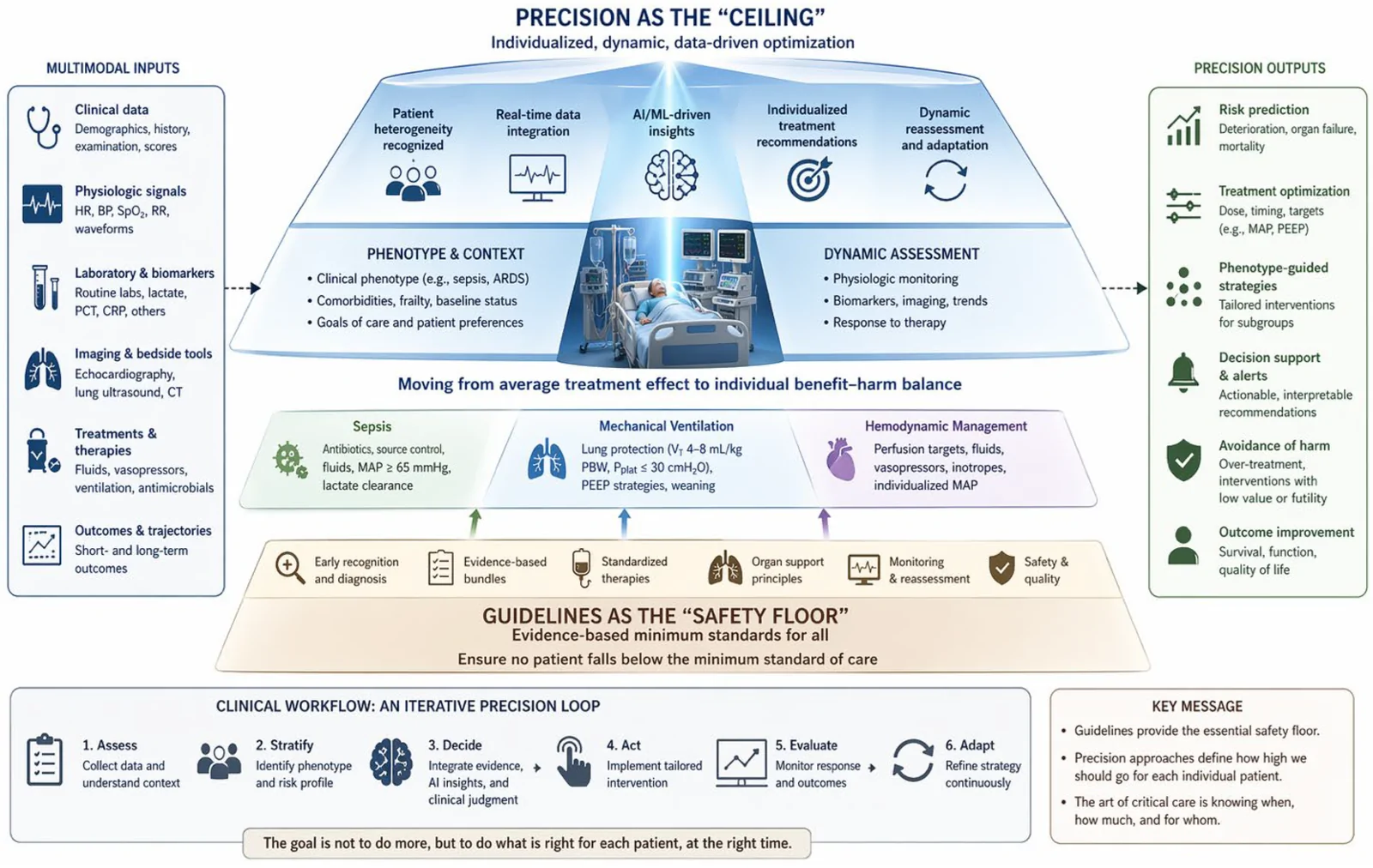
Conceptual framework: Guidelines as the “Safety Floor”, precision medicine as the “Ceiling” in critical care.

## Data Availability

No new data were created or analyzed in this study. Data sharing is not applicable to this article.

## Abbreviations

ICU	Intensive Care Unit
SSC	Surviving Sepsis Campaign
AI	artificial intelligence
SI	Special Issue
RCTs	randomized controlled trials
MAP	Mean Arterial Pressure
ARDS	Acute Respiratory Distress Syndrome
VILI	Ventilator-Induced Lung Injury
PEEP	Positive End-Expiratory Pressure
TBI	traumatic brain injury
EHRs	Electronic Health Records
